# Effect of contrasting phosphorus levels on nitrous oxide and carbon dioxide emissions from temperate grassland soils

**DOI:** 10.1038/s41598-022-06661-2

**Published:** 2022-02-16

**Authors:** Amanuel W. Gebremichael, David P. Wall, Rosie M. O’Neill, Dominika J. Krol, Fiona Brennan, Gary Lanigan, Karl G. Richards

**Affiliations:** 1 Environmental Research Centre, Teagasc, Johnstown Castle, Co. Wexford, Ireland; 2 University College Dublin, Belfield, Dublin 4, Dublin, Ireland

**Keywords:** Biogeochemistry, Ecology, Environmental sciences

## Abstract

Agricultural practices such as repeated fertilization impact carbon (C), nitrogen (N) and phosphorus (P) cycling and their relationships in the plant–soil continuum, which could have important implications for the magnitude of greenhouse gas emissions. However, little is known about the effect of C and N additions under contrasting soil P availability status on nitrous oxide (N_2_O) and carbon dioxide (CO_2_) emissions. In this study, we conducted a field-based experiment that investigated the impact of long-term (23 years) P management (no (P0, 0 kg P ha^−1^), low (P15, 15 kg P ha^−1^) and high (P45, 45 kg P ha^−1^) P inputs) on N_2_O and CO_2_ emissions following two C + N application events in two managed grassland ecosystems with loam and sandy loam soils. The magnitude of fluxes varied between the soil P availability levels. Cumulative N_2_O emission was significantly higher in P0 soils (1.08 ± 0.09 g N_2_O-N m^−2^) than P45 soils (0.63 ± 0.03 g N_2_O-N m^−2^), with the loam soil (1.04 ± 0.04 g N_2_O-N m^−2^) producing significantly higher emissions than the sandy loam soil (0.88 ± 0.05 g N_2_O-N m^−2^). We conclude that P-limitation stimulates N_2_O emissions, whereas P-enrichment promotes soil respiration in these temperate grassland sites. Our findings inform effective nutrient management strategies underpinning optimized use of N and P inputs to agricultural soils as mitigation measures for both food security and reducing greenhouse gas emissions.

## Introduction

Nitrous oxide (N_2_O) and carbon dioxide (CO_2_) are two of the main greenhouse gases (GHGs) emitted from agricultural soils. Nitrous oxide is a potent GHG with 298 times more global warming potential than CO_2_ and arises from application of organic and inorganic nitrogen to soil^1^. Further, carbon dioxide emitted from soil auto- and heterotrophic respiration represents an important flux component in the global carbon cycle between soil and the atmosphere^2^. Grasslands constitute one of the dominant land uses in Europe, comprising 38% of agricultural land, and grassland management practices substantially contribute to GHG emissions^3,4^. Owing to their large capacity for storing soil organic carbon (SOC), grasslands play a significant role in mitigating climate change^5^. Intensively farmed grassland soils are routinely supplemented with nutrient inputs, such as nitrogen (N) and phosphorus (P), to increase herbage biomass production, as these nutrients support plant photosynthesis, protein synthesis, and energy transfer. Despite their importance, imbalanced use or availability of N and P may induce significant alterations in ecosystem structure and functioning, and thereby dynamics of carbon (C) and nitrogen cycles^6^.

Long term and repeated fertilizer applications affect the C:N:P ratio and the cycling of these nutrient in agricultural soils through changes in microbial biomass and community composition^7–10^. Phosphorus and nitrogen fertilization have been found to impact mycorrhizal community and biomass^7,11^, plant species richness and diversity^12^ as well as root exudation and turnover^13^, all of which could influence C movement and plant–soil nutrient relations in ecosystems. Microorganisms can obtain C from organic material and mineralise it into simpler inorganic compounds to release essential nutrients, whose availability in soil limit successful plant and microbial growth. The availability of N and P relative to C (stoichiometric relationships) determines microbial mineralization or immobilization of these nutrients^14,15^ and may strongly affect C dynamics in an ecosystem. Studies suggest strong limitations of N and P on heterotrophic respiration indicating tight coupling of essential nutrients and carbon^16–18^. Nitrous oxide is primarily produced through microbial nitrification and denitrification, and its production via these pathways are affected by microbial composition as well as the availability of soil mineral N and phosphate, C substrate, oxygen, soil moisture, pH, and soil temperature^17,19^. While it is known that N fertilizer applications contribute to the formation of N_2_O^4^, there is a poor understanding of the interaction between soil nutrients and carbon availability, and their subsequent impact on N_2_O and CO_2_ emissions in agricultural soils.

Fertilizer-driven changes in managed grassland soils could have functional implications for the composition of arbuscular mycorrhizal fungi (AMF) and alter their symbiotic relationship with plants^15,20–22^. This symbiotic relationship comprises of increased access to N and P facilitated by the AMF to the plant in return for C^11,23^. However, the extent to which N, P and C is exchanged could be influenced by an increasing use of N and P fertilizers. In a P-rich soil, N enrichment has been found to cause reduced allocation of photosynthates to mycorrhizae arbuscules, coils and extraradical hyphae^23^. In contrast, nitrogen enrichment of low P soils increased C allocations to these structures^23^. Thus, the availability of N relative to P in mycorrhizal system could affect the relationship between fungi and hosting plants and limit the ability of fungi to procure the elements. Lower P levels were associated with a significant increase of AMF colonization in a study conducted in the same experimental field of the current investigation^24^. Contrasting presence of AMF in agricultural soils could have major implications for variable N_2_O and CO_2_ formation and may result in different nutrient use of efficiency of plants. Bender et al.^25^ and Storer et al.^26^ showed reduced N_2_O emissions in soils with abundant presence of fungi group, AMF, despite fungi are generally considered as a source of N_2_O as they lack N2O reductase^27^. In contrast to these findings, Okiobe et al.^28^ demonstrated promoted potential N_2_O production as a result of decreased abundance of arbuscular mycorrhizal fungi. In a recent laboratory-based study, significantly higher N_2_O emission was observed in a P-limited soil than in a P-enriched soil following the same input of C and N in the two varying P-levels^29^. However, this relationship requires further investigation and verification under natural field conditions with plants present. Here we investigate the influence of N fertilizer addition and C availability on N_2_O and CO_2_ emissions across two agricultural soils with sandy loam (Site A) and loam (Site B) textures with differing soil P levels in each soil^30^. We hypothesized that the largest magnitude of N_2_O release occurs at low soil P levels in grassland soils. We further hypothesized that CO_2_ production increases with increasing levels of P in these soils. The main objective was to understand how N_2_O and CO_2_ emissions are affected in response to C + N additions across a soil P gradient.

## Materials and method

### Site description

This experiment was conducted in two long-term P-trial grassland sites (Site A and Site B) situated in proximity (~ 350 m) to each other in the dairy farm at Johnstown Castle, Wexford, Co. Wexford, Ireland (6°49′ W, 52°29′ N). The sites were grazed permanent grasslands before establishment. When the experiment was established in 1995, 16 (10 m × 2 m) plots were formed in each site in a fully randomised block design with four replicates. The two sites established were selected to represent different soil types and drainage classes. Site A is a moderately drained brown earth and site B is an imperfectly drained gley soil^31^. Each year in February, each plot received one of the four phosphorous (P) fertilization rates (16% P superphosphate): 0 (P0), 15 (P15), 30 (P30), and 45 (P45) kg P ha^−1^ year^−1^. All plots were initially sown with *Lolium perenne* and reseeded in 2016 with the same species. However, plant species such as *Poa trivialis*, *Agropyron repens, Trifolium repens* were present to a lesser extent. Above-ground biomass is harvested each month between February and August followed by 40 kg N ha^−1^ fertilizer applications. In the year (2019) of this experiment and the years before, SulCAN as a solid was applied at the first or second week of each month during February-August and potassium (K) as muriate of potash (KCl) was applied in February at a rate of 125 kg K ha^−1^. SulCAN contains 26.7% N in the form of nitric and ammoniacal nitrogen and 5% water soluble Sulphur. For this study plots receiving P0, P15 and P45 at the two field sites were set up to carry out this experiment. The two sites were selected as they had slightly different soil properties and thus there was an opportunity to consider a soil × treatment effect in the experiment.

### Experimental design

Fertilizer N and substrate C were applied on 8 May and 12 June in the experiment undertaken between May and July 2019, which represents the main growing season in Ireland. Within each plot, an area of 1 m × 1 m was selected. Following N fertilizer application (40 kg N ha^−1^) to all plots, carbon substrate [mixture of glucose (40%), sodium acetate (30%) and methanol (30%)] was applied once within the selected area using a sprayer watering can. Labile C available in animal excreta usually contains carbohydrates, volatile fatty acids, and alcohols^32^; as such different carbon substrates were applied to mimic this. Our review of the literature also indicated that C source types could differentially affect denitrifying communities and consequently denitrification rate. Thus, a mixture of three C sources was used to decrease bias of one microbial group over another as a result of single substrate use. Carbon was supplied to alleviate C-limitations of denitrification and nitrification processes as observed by O’Neill et al.^29^ in soils from this trial and to ensure equal substrate availability across all soil P levels. Equivalent C input rate of 0.63 g C m^−2^ day^−1^ was added to represent a daily rate of plant carbon input from *Lolium perenne* dominated ecosystem^33^. Soil samples were collected on eight occasions throughout the experimental period. Soil was sampled from across each selected area to a depth of 10 cm, sieved through 4 mm sieve and analysed for soil mineral N and microbial biomass.

### Soil properties, plant biomass and climate parameters

Physico-chemical soil properties were characterized by taking samples from 10 cm depth from each plot in the two sites before the commencement of the experiment. Soil pH was measured in water (2:1, water volume:soil mass) using Sally pH Auto analyser Dilution System (Gilson 215, Gilson, Dunstable, England). Soil organic matter (SOM) content was determined from mass loss on ignition at 550 °C for 7 h. Total C and total N concentrations were measured using a TrueSpec C/N analyser (TruSpec, LECO Corporation, Michigan, USA). Plant available P, potassium (K), and magnesium (Mg) were estimated using Morgan’s extraction^34^ and analysed using a Lachat QuickChem 8500 Series 2 Flow injection Analyzer (Lachat, QuickChem, 5600 Loveland, Colorado, USA). Particle size analysis was performed using the Pipette method^35^, where 2 mm sieved dry soil (20 g) was pre-treated with 6% H_2_O_2_, 3% NH_4_OH, and 5% sodium hexametaphosphate before separating soil aliquots into particle sizes. Water Holding Capacity (WHC) was determined from the mass difference between water-saturated and then overnight dried (105 °C) soil. Bulk density was determined by dividing weight of oven-dried soil by the total soil volume.

To determine the mineral N concentrations, ten gram fresh soil was extracted with 50 mL 2 M KCl (5:1 solution to soil ratio). The supernatant was filtered through Whatman No. 1 filter paper and filtrates were stored in a cold room at 4 °C for about a week until analysis. Ammonium (NH_4_^+^) and nitrate (NO_3_^−^) concentrations in the extracts were analysed by the Aquakem 600 discrete analyser.

Above-ground plant biomass from each plot of both sites was harvested twice during the experiment period (June 10 and July 11, 2019) to a height of ~ 5 cm using a Haldrup plot harvester. The total harvested biomass weight from each plot was recorded and a 100 g sub-sample was taken for dry matter (DM) analysis. Each fresh herbage sub-sample was weighed and placed in an oven at 70 °C for 3 days, and dry weight of the biomass was determined after re-weighing.

Rainfall records for the experiment period were obtained from a Met Éireann weather observing station located in Teagasc dairy farm in Johnstown Castle, Co. Wexford., situated within a 100 m distance from the experimental sites. Volumetric soil moisture content and temperature was measured to 5 cm depth on individual plots on each gas sampling occasion using a handheld theta probe (WET-2 WET Sensor, Delta-T Devices, Cambridge, England). Water-filled pore space (WFPS) were calculated from the soil moisture values, bulk density of the soils, and soil particle density (2.65 g cm^−3^).

### Microbial biomass, glomalin-related soil protein and potential denitrification activity

Soils were analysed for microbial biomass nitrogen (MBN), phosphorus (MBP) and carbon (MBC) using the fumigation extraction method as described respectively in (Brooks et al.^36,37^, and Vance et al.^38^). Five gram fumigated (24 h) and non-fumigated soil samples were extracted with 100 mL 0.5 M NaHCO_3_ and analysed for P colorimetrically using an Aquakem 600 discrete analyser (Thermo Electron OY, Vantaa, Finland). In order to avoid the spike readings by the instrument due to the effervescent nature of NaHCO_3_, one millilitre of 10% HCl was added to 10 mL extracts and diluted to 50 mL using distilled water. Microbial P was calculated by subtracting the P concentration of non-fumigated samples from fumigated samples, and dividing the result by an extraction factor of 0.4^37^.

Microbial biomass C and N were determined similarly using chloroform fumigation method with extraction period of 48 h with 0.5 M K_2_SO_4_^38^. The extracts of the fumigated and non-fumigated samples were analysed for total C and N using a TOC-L CPH/CPN analyser (Shimadzu, Tokyo, Japan), and the differences, divided by correction factors of 0.45 and 0.54, were used to estimate the microbial biomass C and N, respectively.

Glomalin is a glycoprotein produced by AMF and can be used as an indicator of mycorrhizal colonization in the plant root-soil interface^39^. Total glomalin-related soil protein (GRPS) was extracted by 90 min of autoclaving (121 °C) of 1 g air-dried soil in 8 mL of 50 mM sodium citrate adjusted to pH 8.0 with HCl^40^. Three additional sequential extractions were performed with the sodium citrate solution by autoclaving for 60 min until no red-brown color was visible in the last supernatant. After autoclaving, the samples were centrifuged at 10,000 revolutions per minute (rpm) for 5 min. The amounts of glomalin in the extracts were quantified using the Bradford dye-binding assay with bovine serum albumin (BSA) as the standard (2 mg mL^−1^). In a 96-well plate, replicated 200 µL of standard or extracts and 50 µL of dye reagent were added in each well and mixed using a microplate mixer. The Bradford-reactive substance was determined by measuring absorbance at 600 nm using Microplate Reader (Modulus Microplate Multimode Reader, Turner BioSystems, Sunnyvale, California, USA). Sample concentrations were determined using the standard curve. Potential denitrification activity (PDA) was determined using the acetylene inhibition method, modified from Pell et al.^41^. Briefly, replicated 20 g fresh soils were added into two identical flasks from a sample of soil. The flasks were then sealed with a rubber stopper and flushed and filled with helium after evacuating the headspace air. In one of the replicas, 10% of the headspaces were removed and replaced by acetylene. All flasks were incubated at 15 °C on an orbital shaker at 175 rpm for 30 min followed by the addition of a nutrient solution containing 75 mmol L^−1^ KNO_3_, 37.5 mmol L^−1^ Na-succinate, 25 mmol L^−1^ glucose, and 75 mM Na-acetate. Gas samples were taken from the headspace every 1 h for 5 h. N_2_O concentrations were determined using a gas chromatograph (Bruker, Scion 456-GC, Livingston, Scotland), and PDA was calculated from the rate of change of N_2_O concentrations over time from acetylene amended flasks.

### N_2_O and CO_2_ flux measurements

Gas samples (N_2_O and CO_2_ fluxes) were measured before and after the application of N fertilizer and C substrates, with a daily sampling for 10 days directly after C + N additions and 3–4 times a week in the third and fourth week and 2–3 times a week in the subsequent weeks. A rectangular (40 × 40 cm) static collar, made of stainless steel (opaque), was anchored 5 cm deep into the soil within the marked area of 1 m × 1 m in each of the selected plots. During gas sampling, a 10 cm tall chamber lid fitted with two septa on top was placed on the collar lined with neoprene rubber band. To ensure hermetic sealing of the headspace during sampling, the ring area of the collar was half-filled with water, and a 10 kg weight was placed on the top of the lid to compress the seal. Gas samples were collected between 09:30 and 11:30 local time using a 10 mL Luer lock syringe fitted with a hypodermic needle via one of the septa at 0, 20, and 40 min after chamber closure. Prior to transferring the final sample into a pre-evacuated 7 mL glass vial, air in the chamber headspace was mixed by flushing the syringe three times. Gas samples were analysed using a gas chromatograph (Bruker, Scion 456-GC, Livingston, Scotland) fitted with an electron capture detector to analyse for N_2_O concentrations and a thermal conductivity detector to analyse for CO_2_ concentrations. Daily Fluxes (F) were calculated for each plot using the following equation:

$$ F = \left( {\frac{\Delta C}{{\Delta t}}} \right) \times \left( {\frac{M \times P}{{T \times R}}} \right) \times \left( \frac{V}{A} \right) $$

where ∆C is the change in gas concentration in the chamber headspace during chamber enclosure period in ppbv, ∆t is chamber closing period in minutes, so ∆C/∆t is the slope of the gas concentration with time. M is the molar mass of N_2_O-N (28 g mol^−1^) and CO_2_-C (12 g mol^−1^), P and T are the atmospheric pressure (Pa) and temperature (K). Atmospheric pressure values were obtained from the nearby weather station whereas for T, wet sensor values were used. V is the headspace volume of the closed chamber (m^3^) and A is surface are of the chamber (m^3^). R is the ideal gas constant (8.314 J K^−1^ mol^−1^). Daily flux for each treatment is reported as the average of the replicates.

Cumulative N_2_O and CO_2_ emissions were calculated over each application period by multiplying the daily N_2_O and CO_2_ fluxes by the number of days between two consecutive measurements. A summation of the cumulative flux of each application period is reported as the total cumulative flux.

### Statistical analysis

ANOVAs with repeated measures were used to test for the C + N addition effect on N_2_O and CO_2_ emissions, MBC, MBN, MBP, NO_3_^−^, and NH_4_^+^ with P treatment, site, and day of measurement as fixed effects, and individual plots as a random effect. Two-way ANOVA was applied to test for main and interaction effects of P treatment and site on cumulative N_2_O and CO_2_ emissions, soil property parameters (Table 1), plant biomass, and GRSP. Prior to analysis, response variables were checked for normality (sphericity for repeated ANOVA) and homogeneity of variance, and log transformed when required. Tukey’s HSD post-hoc tests were conducted to identify pair-wise comparisons of significant effects at P < 0.05. We performed Spearman’s rank correlation to assess the correlations between soil biophysicochemical parameters, plant biomass, and N_2_O and CO_2_ emissions.

**Table 1:** Soil properties reported as mean ± SE (n = 4) for each treatment in Site A and Site B.

	Site A	Site B				
Soil classification	Brown earth	Gley				
Sand (%)	58.60	45.60				
Silt (%)	26.80	36.60				
Clay (%)	14.60	17.80				
Bulk density (g cm^−3^)	1.30 ± 0.03^a^	1.28 ± 0.04^a^				
WHC (%)	30.23 ± 0.63^a^	25.36 ± 0.46^b^				

	P0	P15	P45	P0	P15	P45
C (%)	3.61 ± 0.16^a^	3.56 ± 0.06^a^	3.45 ± 0.11^a^	2.86 ± 0.07^b^	2.80 ± 0.01^b^	2.88 ± 0.05^b^
N (%)	0.40 ± 0.02^a^	0.40 ± 0.01^a^	0.37 ± 0.01^a^	0.32 ± 0.01^b^	0.31 ± 0.01^b^	0.30 ± 0.01^b^
SOM (%)	8.63 ± 0.34^a^	8.65 ± 0.03^a^	8.58 ± 0.26^a^	7.08 ± 0.09^b^	7.08 ± 0.05^b^	7.25 ± 0.09^b^
pH	5.83 ± 0.04^bc^	5.84 ± 0.07^bc^	6.05 ± 0.08^ab^	5.68 ± 0.04^c^	5.78 ± 0.13^bc^	6.21 ± 0.09^a^
Morgan’s P (mg kg^−1^)	2.01 ± 0.15^b^	2.31 ± 0.07^b^	6.77 ± 0.08^a^	1.39 ± 0.04^b^	1.58 ± 0.13^b^	5.98 ± 0.09^a^
Morgan’s K (mg kg^−1^)	93.9 ± 7.93^a^	59.7 ± 15.8^ab^	38.6 ± 6.61^ab^	92.1 ± 21.7^a^	37.6 ± 6.85^b^	29.4 ± 2.09^b^
Morgan’s Mg (mg kg^−1^)	74.2 ± 4.86^a^	66.3 ± 3.85^ab^	70.9 ± 5.28^ab^	56.7 ± 1.14^bc^	45.8 ± 1.19^bc^	56.5 ± 3.99^c^

Different superscript letters indicate significant differences (p < 0.05) among phosphorous treatments in the two sites.

ANOVA analysis was performed using lmer function in lme4 package^42^ within R software. All statistical analyses were conducted using R, version 3.6.0^43^.

## Results

### Soil properties and mineral nitrogen

Total C, total N, Mg, and OM were significantly greater in Site A than Site B (P < 0.001) (Table 1). There was a significant effect of phosphorous treatment on soil K, Mg, P and pH (P < 0.001). The pH of P45 was generally higher than the P0 and P15 in the two sites, but the P45 at site B was significantly higher than P0 and P15 (P < 0.01). Expectedly, the P content of P45 was significantly higher than P0 and P15 at both sites (P < 0.01) (Table 1). Site B had significantly greater WHC than Site A.

**Figure 1:**
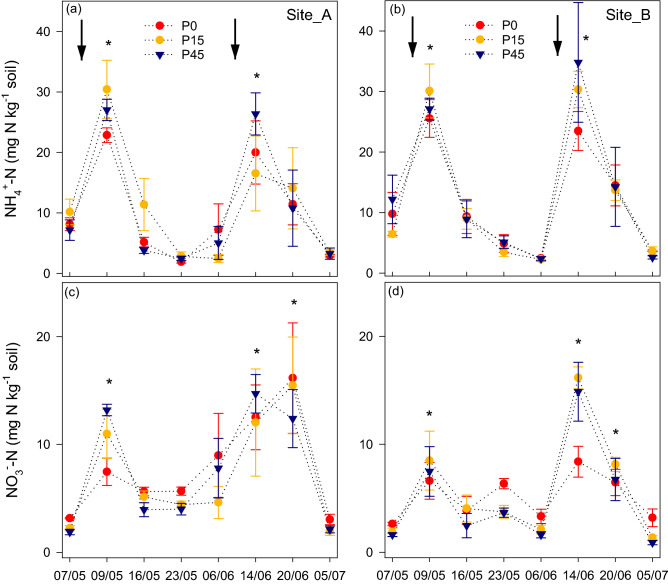
Mean concentrations of soil extractable (**a**) and (**b**) NH_4_^+^-N and (**c**) and (**d**) NO_3_^–^N in site A and site B before and after C + N addition in the long-term P treatments of 0 (P0), 15 (P15) and 45 (P45) kg Pi ha^−1^. The arrows indicate the time of N and C addition. Error bars are standard errors of the mean (n = 4).

C + N addition significantly (P < 0.01) increased soil NH_4_^+^-N and NO_3_^−^-N concentrations in the two sites with the highest increase observed in P45 and P15 plots (Fig. 1). However, the NH_4_^+^-N and NO_3_^−^-N concentrations decreased rapidly in the following week except the NO_3_^−^-N concentrations in site A after the second application when it further increased before decreasing afterward (Fig. 1). Site B had generally significantly higher NH_4_^+^-N concentrations (P = 0.05) (Fig. 1).

### Precipitation, WFPS and soil temperature

**Figure 2:**
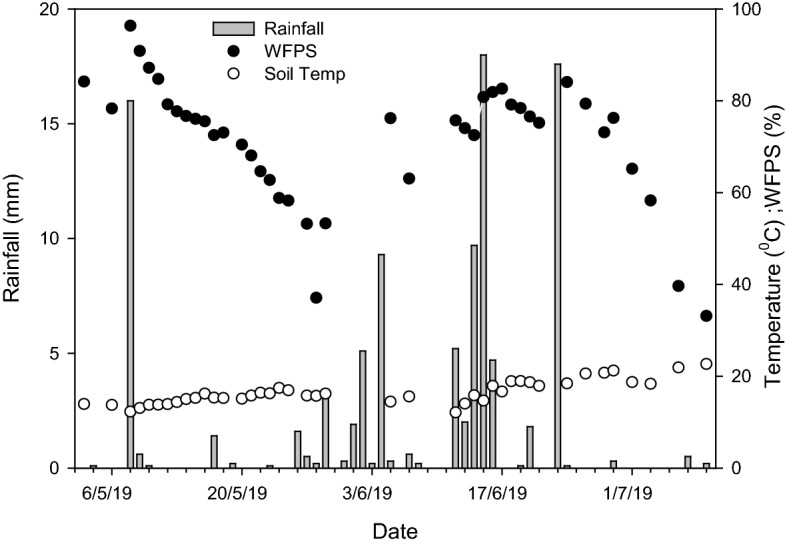
Rainfall, soil temperature and water-filled pore space (WFPS) over the experimental period. Note that soil temperature and WFPS did not differ appreciably between sites and treatments. Therefore, their mean values (n = 6) are presented.

Daily precipitation ranged from 0.1 to 18 mm during the experiment period, with most of the rainfall occurring in the period following the second C + N addition (Fig. 2). Thus, the cumulative rainfall (60.2 mm) during the second addition was greater than the first (41.7 mm), which is considerably dry, compared to the total mean (162.5 mm) of the previous 10 years (2009–2018) of the same period. WFPS decreased progressively from 96.35 to 53.19% following the first addition event, but it stayed above 65.19% for the majority of the second application period (Fig. 2). Owing to a co-occurrence of fertilization events with the preceding rainfall, the addition of C solution did not cause a further increase in soil moisture content. Average soil temperature of 15 °C was recorded in the first fertilization period, which was slightly lower than the second period (18 °C) (Fig. 2).

### N_2_O and CO_2_ emissions

**Figure 3:**
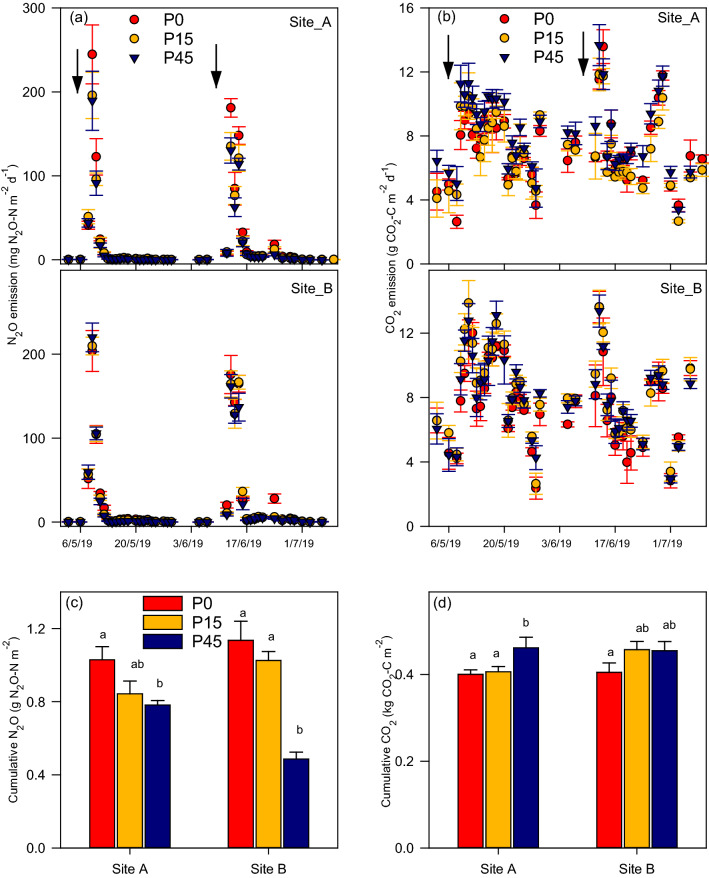
N_2_O (**a**) and CO_2_ (**b**) emissions before and after N fertilizer and C substrate additions and cumulative N_2_O (**c**) and CO_2_ (**d**) emissions in two long-term P trial grassland sites (site A and site B) with phosphorous levels 0 (P0), 15 (P15) and 45 (P45) kg Pi ha^−1^. The arrows indicate the first and the second time of N and C addition. Error bars are standard errors of the mean (n = 4). Letters in (**c**) and (**d**) indicate significant differences (P < 0.05) between P levels.

An increase in labile C and mineral N-availability via the applications of fertilizer and glucose-acetate- methanol mixture to P0, P15, and P45 plots in the two sites resulted in increased N_2_O emissions, reaching a maximum N_2_O flux 2 days after application before decreasing after 8 days to background flux levels for both application events (Fig. 3a). The effect of P-treatment (P = 0.029) and site (P = 0.010) was significant but the interaction of the two was not significant. The emissions associated with the P0 treatment was significantly higher (P = 0.026) compared P45 treatments, indicating that low soil phosphorous enhanced N_2_O production (Fig. 3a). The cumulative N_2_O emission was significantly higher (P = 0.021) in P0 treatment than P45 treatment in both sites (Fig. 3c). The cumulative N_2_O emission in P0 was higher than in P15 treatment in the two sites. The cumulative N_2_O emission in site B (1.04 ± 0.04 g N_2_O-N m^−2^) was significantly higher (P = 0.011) than in site A (0.88 ± 0.05 g N_2_O-N m^−2^) (Fig. 3c). Although peak N_2_O flux occurred after the first C + N addition, the cumulative N_2_O emission following the second addition was significantly higher than that of the first.

Multiple successive peak CO_2_ emissions were observed following C + N addition in all treatments (Fig. 3b). The CO_2_ emission in P45 was higher than in P0 and significantly higher than in P15 (P = 0.036) plots in site A whereas, in site B, the CO_2_ emission in P15 was higher than in P0 plots. There was no significant difference between CO_2_ emissions at P45 and P0 in site B.

There were no significant differences in cumulative CO_2_ emission either between sites or site × treatment interaction (Fig. 3d). However, cumulative CO_2_ emission was significantly higher (P = 0.047) in the P45 and higher in P15 than in the P0 plots (Fig. 3d), being highest overall in the P45. There was no significant variation in the resultant cumulative CO_2_ emission between the first and the second application events, despite generally higher cumulative emission after the first addition.

### Microbial biomass and glomalin-related soil protein (GRSP)

No significant interaction of site and treatment was observed. Total GRSP varied between the two sites with Site B having significantly higher (P < 0.01) glomalin concentrations (Table 2). Total GRSP in P0 treatments of Site A and B were significantly greater than the P15 and P45 treatments (P < 0.01) (Table 2).

**Table 2:** Dry matter (DM) yield (kg ha^−1^), glomalin-related soil protein (GRSP) (mg g^−1^ BSA), and potential denitrification (PDA) (ng N_2_O-N g^−1^ min^−1^) values for each P treatment in Site A and Site B. Letters indicate significant differences (P < 0.05) between P treatments.

Parameters	Site A			Site B		
	P0	P15	P45	P0	P15	P45
DM yield (May–June)	2885 ± 189 b	4018 ± 111 a	3782 ± 146 a	3120 ± 283 b	3839 ± 323 ab	3932 ± 221 a
DM yield (June-July)	1306 ± 131 b	1684 ± 50.6 a	1798 ± 62.1 a	1398 ± 30.4 d	1898 ± 94.4 c	1978 ± 107 c
GRSP	925 ± 42.5 a	868 ± 81.1 b	747 ± 24.1 b	803 ± 31.9 c	729 ± 58.5 bd	678 ± 36.6 d
PDA	4.45 ± 0.43 a	3.52 ± 0.42 b	3.88 ± 0.44 ab	2.58 ± 0.12 c	3.65 ± 0.67 b	2.80 ± 0.48 c

**Figure 4:**
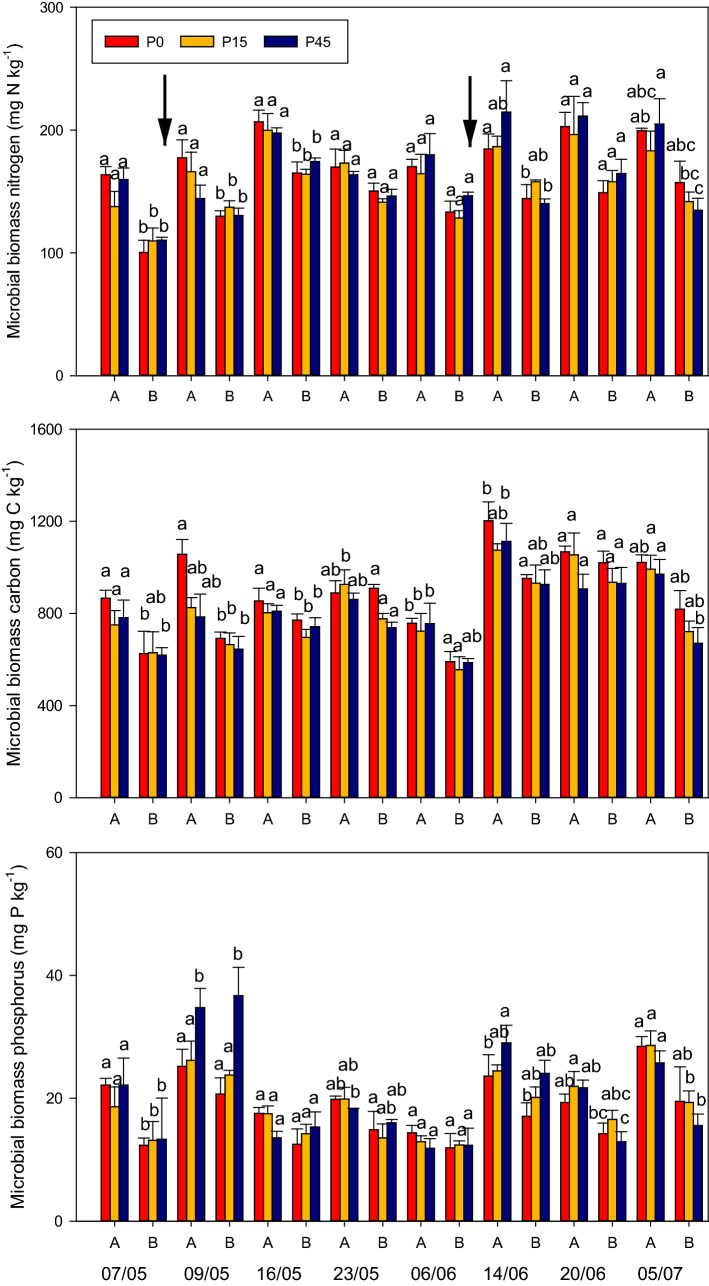
Microbial biomass N, C, and P in site A and site B of the long-term permanent grassland sites with 0 (P0), 15 (P15) and 45 (P45) kg Pi ha^−1^ phosphorous levels. The arrows indicate the time of C + N addition. Error bars are standard errors of the mean (n = 4). Letters indicate significant differences (P < 0.05) between P levels within the same sampling date.

Significant treatment effect was observed in MBN (P = 0.045) and MBP (P = 0.012) following the first and second fertilization, respectively (Fig. 4a,c). Sampling time was a significant factor (P < 0.001) in determining the microbial biomasses whose levels were considerably higher in the first and second sampling after C + N additions. In the first application, MBN was significantly greater (P < 0.010) at site A for every treatment on 07/05, 09/05, and 16/05 in the first application, whereas in the second application MBN at site A was significantly greater (P < 0.010) on 14/06 for P0 and P45 and on 05/07 for P45 treatment (Fig. 4a). Significantly greater MBP at site A was observed on 09/05 and 05/07 at P0 (P = 0.019) and P45 (P = 0.045) treatments, respectively (Fig. 4c). MBC at P0 plots of site A was significantly greater at 09/05 (P = 0.034) and 05/07 (P = 0.030) (Fig. 4b).

### Plant biomass

No interaction of site and treatment was observed for plant dry matter. The dry matter yield at the end of the first C + N addition (May–June) was significantly (P < 0.01) higher than the dry matter at the end of the second application (June–July) (Table 2). There was no noticeable difference in dry matter yield between P15 and P45 plots, but the yield in these plots was significantly greater (P < 0.01) than the yield in P0 in both sites (Table 2). Site B during the second addition had significantly higher dry matter yield than site A at every corresponding phosphorous level plot (P = 0.034).

### Correlations between N_2_O and CO_2_ emissions and soil and plant parameters

The daily N_2_O emissions were significantly correlated with MBP, MBC, NH_4_^+^, NO_3_^−^, and WFPS, but were not correlated with MBN and soil temperature by Spearman’s rank correlation (Table 3). Cumulative N_2_O and CO_2_ emissions were significantly correlated with Glomalin content, which was related to C, K, Mg, N, and plant biomass (Table 4). CO_2_ fluxes were significantly correlated with MBP, NH_4_^+^, NO_3_^−^, and N_2_O (Table 3). A significant positive association was found among PDA, C, and N with the PDA correlated neither with cumulative N_2_O nor CO_2_ emissions (Table 4).

**Table 3:** Spearman’s rank correlation matrix of daily N_2_O and CO_2_ emissions, WFPS, temperature, mineral nitrogen and microbial biomass (N = 192). *p < 0.05; **p < 0.01.

Parameters	1	2	3	4	5	6	7	8	9
1. MBP (mg kg^−1^)	1.000								
2. MBN (mg kg^−1^)	0.263**	1.000							
3. MBC (mg kg^−1^)	0.425**	0.617**	1.000						
4. NH_4_^+^-N (mg kg^−1^)	0.329**	− 0.143	0.082	1.000					
5. NO_3_^–^N (mg kg^−1^)	0.322**	0.220**	0.369**	0.572**	1.000				
6. WFPS (%)	0.189*	− 0.215**	− 0.165*	0.526**	0.273**	1.000			
7. Temperature (°C)	0.091	0.298**	0.323**	− 0.318**	− 0.114	− 0.752**	1.000		
8. N_2_O flux (µg-N m^−2^ day^−1^)	0.555**	0.037	0.353**	0.653**	0.633**	0.345**	− 0.091	1.000	
9. CO_2_ flux (mg-C m^−2^ day^−1^)	0.150*	0.031	− 0.033	0.265**	0.228**	0.064	− 0.094	0.405**	1.000

*MBP* microbial biomass phosphorus, *MBN* microbial biomass nitrogen, *MBN* microbial biomass carbon, *NH*_*4*_^+^ *− N* ammonium nitrogen, *NO*_*3*_^*−*^*-N* ammonium nitrogen, *WFPS* water-filled pore space.

**Table 4:** Spearman’s correlation matrix of cumulative N_2_O and CO_2_ emissions, soil chemical properties, and dry matter yield (N = 24). *p < 0.05; **p < 0.01.

	1	2	3	4	5	6	7	8	9	10	11	12
1. C	1.000											
2. K	0.352	1.000										
3. Mg	0.791**	0.357	1.000									
4. N	0.953**	0.403	0.735**	1.000								
5. SOM	0.830**	0.263	0.723**	0.797**	1.000							
6. P	0.344	− 0.418*	0.438*	0.146	0.412*	1.000						
7. pH	0.091	− 0.317	0.201	− 0.107	0.186	0.750**	1.000					
8. Plant biomass	− 0.387	− 0.717**	− 0.285	− 0.500*	− 0.184	0.406*	0.491*	1.000				
9. Cum.N_2_O	0.290	0.243	− 0.230	− 0.252	− 0.497*	− 0.302	− 0.294	− 0.258*	1.000			
10. Cum. CO_2_	0.280	− 0.546**	− 0.248	− 0.340	− 0.213	0.347	0.158	0.485*	− 0.169	1.000		
11. GRSP	0.404*	0.517**	0.442*	0.435*	0.246	− 0.067	− 0.074	− 0.477*	0.470*	− 0.453*	1.000	
12. PDA	0.427*	− 0.057	0.349	0.416*	0.289	0.188	0.017	0.068	− 0.115	− 0.045	0.197	1.000

## Discussion

Carbon and mineral N availability directly influence soil N_2_O and CO_2_ emissions, but the interaction with different P levels have not been systematically studied, especially under field conditions. In this study, N_2_O and CO_2_ emissions were quantified from two long-term P-trials following C and N addition along a soil P gradient. Our study shows that both N_2_O and CO_2_ emissions increased following co-application of C and N; however, the magnitude of the emissions were constrained by the P level, with the highest N_2_O and CO_2_ emissions associated with P-limited and P-enriched soils, respectively (Fig. 3a–d). These results indicate that P limitation or enrichment can play an essential role in determining N_2_O and CO_2_ emissions in grassland ecosystems. While N fertilization, through increasing soil NO_3_^−^ and NH_4_^+^ concentrations, provided the substrates for nitrifiers and denitrifiers for N_2_O production (Fig. 1), the addition of C may have promoted microbial mineralization of C from these substrates or from soil organic C pools. These findings support our first hypothesis that C + N addition in P-limited soil increases N_2_O production and the second hypothesis that C mineralization is increased at higher soil P levels.

### Differences in N_2_O emissions at different P levels

The differences in the N_2_O emission are most likely to be associated with induced differences in the composition, activity and/or diversity of microbial communities in relation to different P levels. Our results suggest the greater N_2_O emission in P-limited soils (Fig. 3a,c) may be associated with higher abundances of arbuscular mycorrhizal fungi (AMF), as indicated by higher levels of glomalin (Table 2), and this may be partially responsible for the difference in N_2_O emissions. The high levels of AMF may have decreased the relative abundance of denitrifying organisms that are capable of reducing N_2_O into N_2_^28^, leading to higher N_2_O emission in P-limited plots. Glomalin is regarded as a metabolite of AMF, and its concentration is largely associated with the abundance of the AMF hyphae^39,40^. In this study, greater concentrations of glomalin were detected in P0 than P15 and P45 treatments indicating that AMF are more pronounced in plots with lower soil P availability. Typically higher MBC in P0 than P15 and P45 in both sites suggest that more carbon could be immobilized by microorganisms (Fig. 4). This may also be indicative of higher abundances of arbuscular mycorrhizal fungi. The significant correlations between daily N_2_O fluxes and MBP and MBC (Table 3) and between cumulative N_2_O emissions and glomalin content (Table 4) support the argument that microbial acquisition of C and P as well as AMF abundance were related to production of N_2_O. Arbuscular mycorrhizal fungi are recognized for their greater C assimilate demand and sink strength and thus may have had a role in the differences observed in MBC^21,44,45^. Owing to the ability of AMF to acquire immobile soil P and trade P for plant growth; they form mutually beneficial relationships with their host to satiate their C demand in P-limited soils^20^. This is in line with previous evidence that showed enhanced AMF colonisation was observed at low P in the experimental plots at Site A^10,24^. Recent findings of Okiobe et al.^28^ showed a strong influence of AMF presence on promoting potential N_2_O production via an increase in hyphal density and via enhanced water stable soil aggregates, indicating possible lack of N_2_O reductase in the denitrification process. However, there are earlier studies that reported contrasting results where AMF reduced N_2_O emissions as a result of reduced soil NH_4_^+^ availability because of enhanced transport of NH_4_^+^ by AMF to the host plant^26,27^. Our observations showed adequate availability of NH_4_^+^ following the C + N application (Fig. 1a,b), thus we can deduce that N_2_O may not be reduced as there was surplus substrate to facilitate denitrification.

The peak N_2_O emissions (Fig. 3a) coincided with higher WFPS, which was above 80% at the time of fertilization (Fig. 2). Higher WFPS coupled with higher available C, being served as donor electron to the denitrifiers could have stimulated denitrifying microorganisms via enhanced anaerobic conditions. While this could be true for all P treatments, the varying magnitude of N_2_O emissions could have resulted partially from the indirect effect of variable heterotrophic respiration due to differences in P availability. High P has been found to impede heterotrophic respiration in grassland ecosystems^16,17^. Similarly, O’Neill et al.^29^ showed higher heterotrophic respiration in the low P than high P in an incubation experiment conducted in the same experimental site utilizing sieved soils with no plant respiration component. Lowering of soil O_2_ concentration as a result of heterotrophic respiration might have promoted more suitable denitrifying conditions in the P-limited soils leading to higher denitrification-derived N_2_O production.

The significant positive correlations between NH_4_^+^ and NO_3_^−^ and N_2_O emissions (Table 3) suggest that N_2_O fluxes depend on the amount of mineral N available in the soil. However, the high N_2_O emission observed in site B (Fig. 3a) in all corresponding treatments might be associated with the higher soil NH_4_^+^ (Fig. 1a,b), particularly evident following the second C + N addition event when the NH_4_^+^ concentration at site B was approximately double the amount in site A. It is subsequent to the second application where the greatest differences and the highest cumulative N_2_O emissions occurred (Fig. 3c). Optimum soil moisture conditions (Fig. 2) and sufficient availability of NH_4_^+^ might have formed more conducive conditions for nitrification in site B. Certainly, denitrification-related N_2_O emission could also be stimulated because equally high NO_3_^−^ concentrations (Fig. 1c,d) were detected in site B. Approximately equal correlation of N_2_O flux with NH_4_^+^ and NO_3_^−^ (Table 3) indicate that both nitrification and denitrification can be important pathways of N_2_O emissions in the two sites. Microbial parameters including MBN, MBC, and MBP showed significantly lower values in site B (Fig. 4) that might suggest that more immobilization of N appears to occur in site A (Fig. 4). These microbial biomass differences might also suggest disparities in the microbial community between the two sites where a subset of the microbial group could have inherently different N-transformation pathways towards regulating N_2_O production. Unexpectedly, the PDA at site A for P0, P15, and P45 was 1.7, 0.9, and 1.4 times that of site B, respectively (Table 2), confirming the presence of different genetic capacity to denitrify and also suggesting higher PDA does not necessarily guarantee higher N_2_O emission in the field. Potential denitrification was related to soil C and N (Table 4), which are significantly higher in site A than site B (Table 1). Climate parameters may have a diminishing role in explaining site differences due to that the two study sites are proximate, and have been under identical management practices. WHC in site A was higher than site B (Table 1). Wang and Cai^46^ observed increasing N_2_O production with increasing WHC.

The soil P level and its effect on greenhouse gas quantifications are usually unaccounted for in almost all ecosystems. This is one of the few field studies demonstrating the relationship between C, N, and P, and the impact on N_2_O emissions from grassland ecosystems. Nonetheless, further studies on the long-term interaction of C, N, and P in multiple ecosystems (soil) types under natural conditions are needed to critically appraise the influence of contrasting P fertilization on N_2_O and CO_2_ emissions. To expand our insight into the role of soil P in ecosystem N cycling, future studies should focus on revealing the effect of variable P content on N transformation pathways, and their linkage to microbial community and specific functional genes. Understanding the effect of soil P on N_2_O emissions may pave the way forward to an optimised use of P and N as mitigation measures that both underpins food security and reducing greenhouse gas emissions.

### Effect of C + N addition on CO_2_ emissions

Unlike the N_2_O emissions, P-enriched plots, relative to the low P, showed greater CO_2_ emission following C + N addition in the long-term grassland sites (Fig. 3d). These variations could be due to the enhancement of autotrophic (root) respiration caused by P availability in the P-enriched plots^16^ and reduced presence of AMF in these plots. Several studies reported increased soil respiration in response to P addition^16,47,48^ because of the stimulating effect of P on aboveground and belowground biomass^16,48^. Higher aboveground plant biomass was generally observed in the P-enriched plots (Table 2) supporting the inference that net primary production and hence autotrophic respiration was higher at high P, which is supported by the positive correlation between cumulative CO_2_ emission and dry matter yield (Table 4) with a proposition that above- and below-ground biomass follow an isometric pattern. Ren et al.^16^ found an annually increasing trend in root biomass and concurrently increasing autotrophic respiration induced by P fertilization in a 4-year field study in an alpine grassland. In a study performed in vivo, Del-Saz et al.^49^ showed a decrease of root respiration via alternative respiratory pathways resulting from decreasing root exudation of carboxylates such as citrate and malate as a result of AMF colonization, which is typically initiated by P-deficiency. This demonstrates the effect of P availability on respiratory pathways, hence soil respiration. Phosphorous plays an important role in the synthesis of nucleic acid and membrane, and enzymatic activations, and sufficient P could have supported specific root respiration^23^. However, aboveground plant respiration can also significantly contribute to the total ecosystem respiration in grassland ecosystems^50^. We found no relationship between CO_2_ emissions and WFPSs and temperature in the two sites (Table 3).

Arbuscular mycorrhizal fungi are capable of acquiring considerable amount of N and P from soil by expanding the surface area of the root system and transport these nutrients to their host plant in exchange for photosynthetically-fixed carbon^20^. The higher glomalin concentration at the P0 (Table 2) indicated higher AM-related C input into soil, which is demonstrated by the positive relationship between glomalin and soil carbon in Table 4. This contributes to macro-aggregate formation and SOM stabilisation^51^. The positive relationship between GRSP and soil carbon in this study is in line with previous findings^52–54^ where positive contributions of glomalin to maintaining the soil carbon pool have been reported. Clemmensen et al.^52^, in their study on boreal forests, using a combined technique of pyrosequencing of DNA-barcodes and isotopes, identified root-related fungi as important regulators of ecosystem C dynamics. Zhang et al.^55^ showed the regulatory power of AMF on soil respiration as AM inhibition resulted in accelerated soil respiration due to increased availability of root-exudated carbohydrate to other microbes in the rhizosphere. These findings, together with ours, suggest that AMF may influence soil C dynamics by increasing SOC recalcitrance either via aggregation or increasing decomposition resistant C species (glomalin, chitin, etc.). This argument is supported by the significant negative correlation between glomalin and cumulative CO_2_ emission (Table 4), such that long-term plant fungi partnership at P0 has increased aggregate stabilization of C via glomalin, causing reduced CO_2_ production. Greater allocation of biomass to roots delivers C to the soil and the greater the depth that rooting occurs, the lower the decomposition due to low redox potential. This can lead to the conclusion that AMF-derived C contributes more to the stable soil C pool than the carbon derived from aboveground dry matter yield (Table 2). Aboveground biomasses in P15 and P45 plots were greater than P0 (Table 2). This is obviously due to a change in C:N:P stoichiometry caused by variable phosphorous supply. In the P0 plots, where there is a limited supply of P, plants form nutritional symbiosis leading to high C-nutrient exchange with AMF, whereas in the P-rich plots such symbiotic relationship is eliminated or reduced due to the repeated N and P fertilization. Where N and P are sufficiently available, plants invest less in mycorrhizas and AMF, instead adjusting C allocation to the aboveground biomass^20,23^. Therefore, these results underscore the need to account for soil C sequestration and C fixation by plants, in addition to CO_2_ fluxes, in order to assess the impact of phosphorus fertilization on C balance of grassland ecosystems and suggest mitigation options, which is achieved through evaluation of changes in SOC over an extended time and assessment of net ecosystem exchange.

## Conclusions

Soil P plays an important role in determining N_2_O and CO_2_ emissions under equivalent C + N conditions. This is the first field study that shows a significant effect of differing soil P levels on the two major greenhouse gases such as N_2_O and CO_2_ in temperate grassland ecosystems. Higher N_2_O emission was observed in P-limited soils whereas P-enrichment enhanced CO_2_ emissions in the two permanent grassland ecosystems. P fertilization can reduce N_2_O emissions derived from N-fertilization but increase CO_2_ emissions.. These findings are important in informing effective management strategies to agronomic practices underpinning an optimized use of N and P as mitigation measures for both food security and reducing greenhouse gas emissions. Furthermore, our findings highlight the need for representation of P in process-based land models with its effect on the dynamics of greenhouse gases in terrestrial ecosystems. Future studies may reveal how the interaction of C and N with P affect specific N-transformation pathways, C sources of mineralization, and microbial communities and their functional traits in these ecosystems.
